# Better together: nanoscale co-delivery systems of therapeutic agents for high-performance cancer therapy

**DOI:** 10.3389/fphar.2024.1389922

**Published:** 2024-05-20

**Authors:** Liyan Sun, Zhe Li, Jinshuai Lan, Ya Wu, Tong Zhang, Yue Ding

**Affiliations:** ^1^ School of Pharmacy, Shanghai University of Traditional Chinese Medicine, Shanghai, China; ^2^ The MOE Innovation Centre for Basic Medicine Research on Qi-Blood TCM Theories, Shanghai University of Traditional Chinese Medicine, Shanghai, China; ^3^ State Key Laboratory of Integration and Innovation of Classic Formula and Modern Chinese Medicine, Shanghai University of Traditional Chinese Medicine, Shanghai, China

**Keywords:** nanoscale co-delivery system, cancer, drug-loading strategies, controlled/targeted delivery, combined therapy

## Abstract

Combination therapies can enhance the sensitivity of cancer to drugs, lower drug doses, and reduce side effects in cancer treatment. However, differences in the physicochemical properties and pharmacokinetics of different therapeutic agents limit their application. To avoid the above dilemma and achieve accurate control of the synergetic ratio, a nanoscale co-delivery system (NCDS) has emerged as a prospective tool for combined therapy in cancer treatment, which is increasingly being used to co-load different therapeutic agents. In this study, we have summarized the mechanisms of therapeutic agents in combination for cancer therapy, nanoscale carriers for co-delivery, drug-loading strategies, and controlled/targeted co-delivery systems, aiming to give a general picture of these powerful approaches for future NCDS research studies.

## Graphical Abstract

**Figure:**
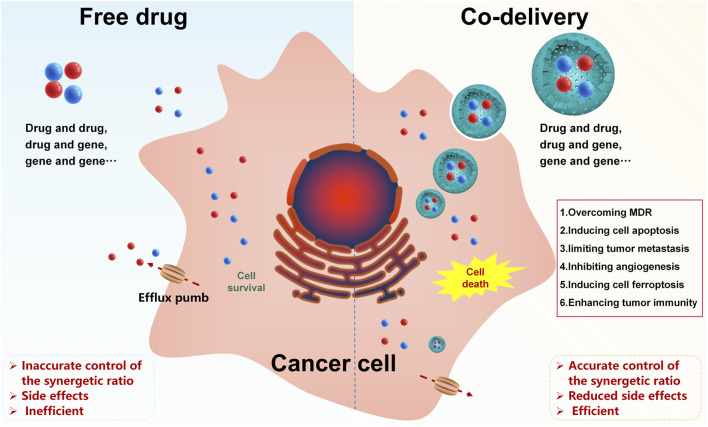
Nanoscale co-delivery systems of therapeutic agents for high-performance cancer therapy.

## 1 Introduction

Conventional mono-therapeutic agents are common in clinical cancer treatment. Hundreds of therapeutic agents have been discovered or synthesized to fight against various cancers. However, the outcomes of clinical mono-therapeutic agents for cancer treatment are usually unsatisfactory due to the physiological complexity of cancers. Moreover, mono-therapeutic agents may usually cause tumor drug resistance. Consequently, researchers have put forward the concept of combination therapy for cancer treatment, which refers to the therapeutic agents co-administrated for the treatment of cancer (Qin et al., 2018). In 1965, Frei et al. first used methotrexate, 6-mercaptopurine, vincristine (VCR), and prednisone in combination for treating acute leukemia, which was proven successful (Frei et al., 1965). Since then, researchers have focused on investigating combination therapies that modulate different signaling pathways in cancer cells, thus sensitizing cancer responses to drugs, lowering drug doses, and reducing side effects. In short, equal or better treatment benefits can be obtained at lower doses when adopting the combination therapy strategy (Bayat et al., 2017). To evaluate the effect of therapeutic agents used in combination, the combination index (CI) has been introduced, where CI < 1 represents a synergistic effect, CI = 1 represents an additive effect, and CI > 1 represents an antagonistic effect (Qin et al., 2018).

Although combination therapies contribute to better cancer treatment to some extent, current combination therapies also have many shortcomings: owing to heterogeneity in the physicochemical properties and pharmacokinetics of diverse therapeutic agents, the amount of these therapeutic agents actually reaching the tumor site differs greatly when the prescribed dose is administered, making it difficult to achieve accurate control of the synergistic ratio. When some therapeutic agents need to be released in a certain time sequence or program, the time control of the therapeutic agent combination is complicated and patients’ compliance is poor. Therefore, there is a pressing need for developing a delivery strategy to homogenize the process of multiple therapeutic agents *in vivo*.

As demanded, a nanoscale co-delivery system (NCDS) has emerged as a potential strategy for drug combination therapy in cancer, which has the advantages of loading therapeutic agents with different physicochemical properties into the same carrier, homogenizing the fate of co-delivered therapeutic agents *in vivo* for a final desired synergistic ratio at the tumor site, as well as reducing toxicity (Mujokoro et al., 2016; Huo et al., 2020). In recent years, the NCDS has been explored to co-load different therapeutic agents, such as drug and drug, drug and gene, or gene and gene co-loading (Chang and Bae, 2011; Creixell and Peppas, 2012; Kang et al., 2015; Qi et al., 2017; Li et al., 2018)^.^ This review mainly discusses the mechanisms of therapeutic agents in combination for the treatment of cancer, nanoscale carriers for co-delivery, drug-loading strategies, and controlled/targeted co-delivery systems, aiming to provide a general picture of these powerful approaches for future NCDS research studies.

## 2 Different mechanisms in nanoscale co-delivery of different therapeutic agents for cancer therapy

**FIGURE 1:**
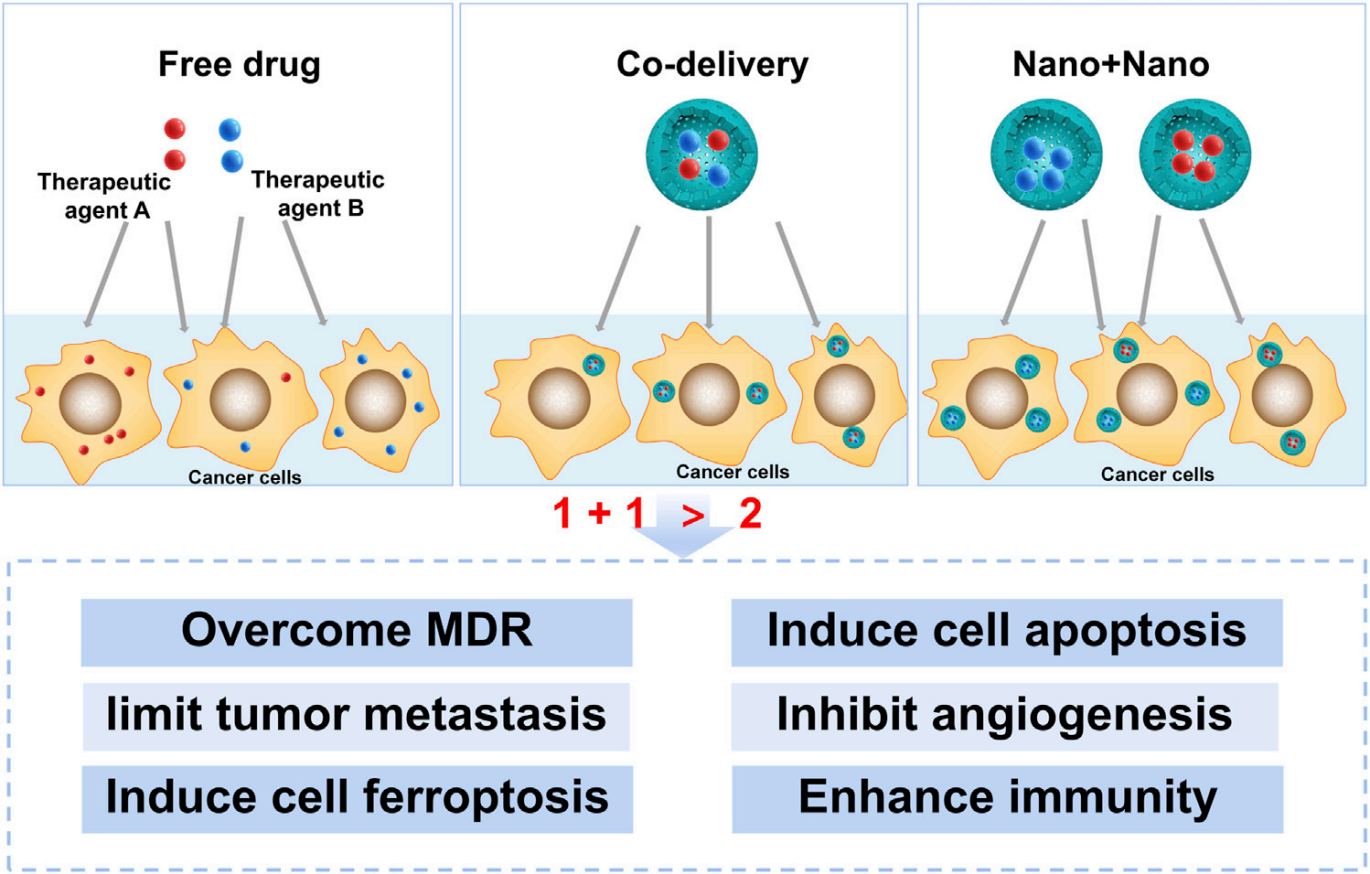
Different mechanisms of co-delivery systems for cancer therapy.

It is profound to understand the mechanisms of different therapeutic agent combinations at the cellular and molecular levels for discovering new combinations and new combination strategies. The mechanisms involved in the NCDS for cancer therapy, including overcoming multidrug resistance (MDR), inducing cell apoptosis, limiting tumor metastasis (invasion), inhibiting angiogenesis, inducing cell ferroptosis, and enhancing antitumor immunity, have been summarized below (Figure 1).

### 2.1 Overcoming MDR in cancer therapy

MDR is a leading obstacle to effective cancer treatment. The main mechanisms for MDR lie in increased detoxification enzyme levels, abnormal uptake and efflux of drugs, drug target enzyme alterations, intracellular redistribution, enhanced DNA repair, and imbalance of survival/apoptosis signaling pathways. Among them, overexpression of drug efflux proteins, such as P-glycoprotein (P-gp), is thought to be the most important reason for MDR (Zong et al., 2019). Various strategies have been implemented to overcome MDR. The co-delivery of different therapeutic agents by nanoparticles (NPs) may be a good strategy to overcome MDR (Mohammad et al., 2018a; Mohammad et al., 2020). The co-delivery of different small molecular chemotherapeutic drugs is most commonly used to overcome MDR. For example, co-loading, accurate ratiometric control of doxorubicin (DOX) and adjudin (ADD, a P-gp inhibitor), and programmed drug release were realized by using poly (b-amino ester)-g-b-cyclodextrin (PBAE-g-b-CD), thus significantly inhibiting the growth of MCF-7/ADR cells (Wang et al., 2019). In another research, a pH-sensitive liposome (pHSL) formulation simultaneously loaded with tariquidar (TQR, a third-generation P-gp inhibitor) and DOX was constructed, and the pHSL/TQR/DOX system could restore the drug sensitivity of OVCAR8/ADR cells. A combination of photodynamic therapy (PDT) agents and chemotherapeutic drugs also provides a way of reversing MDR. A reduction-sensitive NCDS loaded with the near-infrared (NIR) fluorophore (DEB-BDTO) and TQR within a polymeric prodrug (DEB/TQR@PMP micelles) was developed. The micelles exhibited a distinguished synergistic effect on SKOV-3 cells and SKOV-3/MDR cells and inhibited tumor growth *in vivo* when compared to DEB-BDTO or TQR alone. In recent years, silencing MDR genes with small siRNAs has also been considered a powerful tool for reversing MDR. In the research, generation 4 polyamidoamine (G4PAMAM) conjugated with a polyethylene glycol (PEG)-phospholipid copolymer could self-assemble into a micellar nano-preparation and load siRNA (siMDR-1) onto PAMAM moieties and DOX into the lipid hydrophobic core. The combination nano-preparation effectively downregulated P-gp in A2780/ADR and MCF7/ADR cells and reversed the resistance toward DOX (Pan et al., 2019).

Understanding the mechanisms of different therapeutic agent combinations and the various pathways involved in reversing MDR is essential for successful cancer treatment. In addition to the co-delivery of therapeutic agents with siRNA or new-generation potent P-gp inhibitors mentioned above, co-delivery of therapeutic agents with natural alkaloids (such as tetrandrine, quercetin (QUE), kaempferol, and icaritin) may be a good strategy when facing the failure of first- and second-generation P-gp inhibitors (such as verapamil, cyclosporine A, and dexverapamil) (Joshi et al., 2017; Kumar and Jaitak, 2019).

### 2.2 Inducing cell apoptosis in cancer therapy

Apoptosis is programmed cell death that is initiated after damage to DNA and cell organelles, such as the mitochondria and endoplasmic reticulum (Mortezaee et al., 2019). Apoptosis of tumor cells can halt the progression of cancer. Co-delivery of different therapeutic agents by NPs provides a potential strategy to induce cell apoptosis in cancer therapy. To enhance HepG2 cell apoptosis, liposomes loaded with cisplatin (CDDP) and curcumin (CUR) (CDDP/CUR-Lip) were prepared (Cheng et al., 2018). Annexin V-FITC/PI double staining showed a higher percentage of early and late apoptotic cells in CDDP/CUR-Lip when compared with the use of CDDP or CUR alone, which demonstrated that CDDP/CUR-Lip could enhance the cell apoptosis effect. *In vivo* TUNEL assays also showed that tumors in the CDDP/CUR-Lip group had the largest proportion of apoptotic tumor cells. The combination of chemotherapy and photothermal therapy (PTT) in NPs has also emerged as a prospective strategy to induce cell apoptosis in cancer therapy. In this research, a multifunctional pH/reduction dual-responsive drug delivery system loaded with DOX and the photosensitive agent ICG was developed. The polymeric micelles could effectively enhance cellular uptake, cell apoptosis, and toxicity toward BEL-7404 cells and showed a superior anti-tumor effect against tumor-bearing mice when compared with free drugs (Zhang et al., 2018).

### 2.3 Limiting tumor metastasis in cancer therapy

Metastasis mainly includes the following processes: 1) tumor cells leak from the primary tumor site. 2) The leaked tumor cells travel to a distant site via the circulatory system. 3) The colonized tumor cells establish a secondary tumor (Shen et al., 2013). The metastasis of breast cancer is the leading cause of cancer death in women, and the lung is a common location for a secondary tumor that has metastasized from the primary-source tumor (Shen et al., 2013), which impedes successful chemotherapy for cancer (Li et al., 2017). P85-PEI/TPGS (co-delivering twist shRNA (shTwi) and paclitaxel (PTX) with the conjugate of pluronic P85 and polyethyleneimine (PEI) (P85-PEI)/D-a-tocopheryl polyethylene glycol 1,000 succinate (TPGS) complex NPs, (PTPNs)) complex NPs could realize enough cellular uptake and RNA interference on 4T1 cells. The IC_50_ value of PTPNs against 4T1 cells was 63-fold lower than that of free PTX. The downregulation of twist RNA contributed to the significant inhibitory effect of cell migration and invasion. PTPNs also showed prolonged circulation and increased accumulation in the lungs and tumors. *In vivo* antitumor efficacy showed that PTPNs could not only effectively inhibit tumor growth but also completely restrict pulmonary metastasis. In another study, QUE (an Akt inhibitor) in combination with docetaxel (DTX, a chemotherapeutic agent) was loaded with HA/polylactic-co-glycolic acid–polyethyleneimine NPs (PP-HA/NPs) using a modified emulsion solvent evaporation technique. Biodistribution assay showed PP-HA/NPs could accumulate in the tumor and lungs, which meant that PP-HA/NPs could be an effective NCDS to treat metastatic breast cancer (Li et al., 2017). Moreover, Mohammad et al. developed self-assembled nanodrugs with a high drug loading comprising a berberine derivative (Ber) and DOX, which successfully altered the target location of DOX from the nucleus to mitochondria and therefore inhibited the proliferation, invasion, and migration of MDA-MB-231 cells by triggering cell apoptosis (Mohammad et al., 2018b; Mohammad et al., 2019).

### 2.4 Inhibiting angiogenesis in cancer therapy

Angiogenesis plays an important role in tumor growth and progression, which makes anti-angiogenic therapy a particularly appealing antitumor treatment strategy (Gong et al., 2018). As is known to all, vascular endothelial growth factor (VEGF) is vital for inducing angiogenesis. Therefore, anti-angiogenesis by interfering with VEGF could pave the way for efficient cancer therapy (Wang et al., 2017). An amine-functionalized silica NP with combined angiogenesis therapy for breast cancer was successfully developed for co-delivery of angiostatin (ANG) plasmid and candesartan (CD) into MCF-7 cells. The NCDS had a stronger inhibitory effect on angiogenesis *in vitro* by downregulating VEGF expression via different pathways. *In vivo* investigations on MCF-7-bearing mice confirmed that the NCDS exerted superior antitumor efficacy through a synergistic anti-angiogenic mechanism (Gong et al., 2018). Hypoxia-inducible factor (HIF), as an angiogenic master switch, also plays an important role in angiogenesis. Co-delivery of chemotherapy drugs and siRNAs with the effect of inhibiting angiogenesis can promote antitumor therapy. Lipid-polymer hybrid NPs loaded with si-HIF1α and gemcitabine (GEM) were constructed to investigate their synergistic anti-tumor effects. The NCDS could co-deliver si-HIF1α and GEM into panc-1 cells, thus inhibiting the expression of HIF1α and showing an excellent ability of suppressing tumor metastasis. Co-delivery of chemotherapy drugs and other drugs suppressing HIF1α expression promotes anti-tumor therapy.

### 2.5 Inducing cell ferroptosis in cancer therapy

Ferroptosis is a newly proposed non-apoptotic programmed cell death process, showing great potential in cancer therapy (Xu et al., 2019). The combined application of a ferroptosis-based multimodal treatment strategy provides a more reliable new concept for malignant tumor therapy. Ferroptosis can be combined with chemotherapy. Cheng et al. designed a CDDP prodrug-loaded manganese-deposited iron oxide nanoplatform (Pt-FMO). In the nanoplatform, manganese, iron ions, and Pt-drugs could be released from the Pt-FMO in the tumor microenvironment. Manganese catalyzes the Fenton reaction, Pt-drugs promote the generation of H_2_O_2_ in cells, and the Pt-FMO significantly strengthens the catalysis of the Fenton reaction. Moreover, the Pt-drugs would eventually function as CDDP and induce cancer cell apoptosis (Cheng et al., 2021). Ferroptosis can also be combined with sonodynamic therapy (SDT)/phototherapy. Hemoglobin (Hb) was connected with chlorin e6 (Ce6) to construct a 2-in-1 nanoplatform loading sorafenib (SRF@Hb-Ce6), which combined oxygen-boosted PDT and potent ferroptosis (Xu et al., 2020).

### 2.6 Enhancing antitumor immunity

Tumor immunotherapy represents a therapeutic approach that employs immunologic strategies and techniques to potentiate the host’s immune defenses, thereby eliciting an immunogenic assault against tumor cells (Jin et al., 2024). Co-delivery systems in the context of tumor immunotherapy are designed to concurrently transport multiple therapeutic agents, such as antigens, adjuvants, and immunomodulatory drugs, to the tumor microenvironment or immune effector cells, synergistically enhancing the antitumor immune response by orchestrating various aspects of the immune system. The effects of co-delivery on tumor immunity mainly include the following: 1) enhanced antigen presentation: co-delivery systems can enhance the presentation of tumor antigens to immune cells, thereby improving the activation of T cells and the initiation of a more effective immune response against tumors. 2) Improved immune cell recruitment: these systems can also modulate the tumor microenvironment to favor the recruitment of immune cells, such as dendritic cells (DCs), T cells, and natural killer (NK) cells, to the tumor site. 3) Stimulation of immune responses: by delivering adjuvants alongside antigens, co-delivery systems can stimulate stronger and more specific immune responses. Adjuvants help in activating immune cells and in the production of cytokines, which are crucial for a potent antitumor response. 4) Reduction in immune suppression: tumor environments often suppress immune responses. Co-delivery of immunomodulatory agents can counteract this suppression, enabling the immune system to more effectively attack tumor cells. Xing et al. (2022) encapsulated the immunomodulator resveratrol into chitosan-coated liposomes, creating a novel nanoparticle-based carrier (QCS-Res-LP), and attached the protein ovalbumin as a model antigen to its surface. QCS-Res-LP particles showed high encapsulation and adsorption efficiencies for resveratrol and the antigen, respectively. These particles significantly enhanced the uptake of both the antigen and resveratrol by dendritic cells, outperforming standard resveratrol liposomes by a substantial margin. Furthermore, QCS-Res-LP stimulated immune cells to express key surface markers and cytokines without causing damage to red blood cells. Mice immunized with the QCS-Res-LP system containing ovalbumin exhibited a strong immune response, evidenced by elevated levels of specific antibodies and cytokines, indicating a balanced Th1/Th2 response. The study confirmed that the QCS-Res-LP system was an effective method for co-delivery of antigens and immunomodulators, leading to potent and lasting immunity. An IONP-C/O@LP nanocarrier, encapsulated with a lipid membrane, was engineered for co-administration of peptide antigens and adjuvants (CpG DNA) into the antigen-presenting dendritic cells via concurrent membrane fusion and endosomal pathway internalization (Meng et al., 2022). This dual-entry mechanism potentiated the nanovaccine’s capacity to dendritic cells. Furthermore, the iron oxide core contributed to the adjuvant effect by eliciting the production of intracellular reactive oxygen species and augmenting dendritic cell maturation. The preferential localization of IONP-C/O@LP within the dendritic cells of sentinel lymph nodes substantially amplified the population of antigen-specific cytotoxic T lymphocytes within the tumor microenvironment and splenic tissue, culminating in the suppression of tumorigenesis and enhancement of host survival rates. Additionally, empirical evidence substantiated the versatility of this nanovaccine as a delivery platform for clinically pertinent peptide antigens derived from human papilloma virus 16, eliciting specific immune responses *in vivo*.

Combination therapy has become a major trend in the treatment of cancer because of its significant advantages in improving therapeutic effects, reducing toxicity, overcoming MDR, inducing cell apoptosis, limiting tumor metastasis, inhibiting angiogenesis, inducing cell ferroptosis, enhancing antitumor immunity, and so on. The general principles for choosing therapeutic agents are as follows: first of all, one of the basic principles of clinical therapeutic agents for combination therapy is that the therapeutic agents should have different mechanisms in cancer therapy. Drugs with the same pharmacological effects will compete for the site of action and produce pharmacological antagonism. Second, the combination of the therapeutic agents should demonstrate a synergistic therapeutic effect, such as a clinically acceptable combination regimen. In addition, therapeutic agents for combination therapy should not increase the adverse effects on normal tissues.

## 3 Different nanoscale carriers for co-delivery of therapeutic agents

**FIGURE 2:**
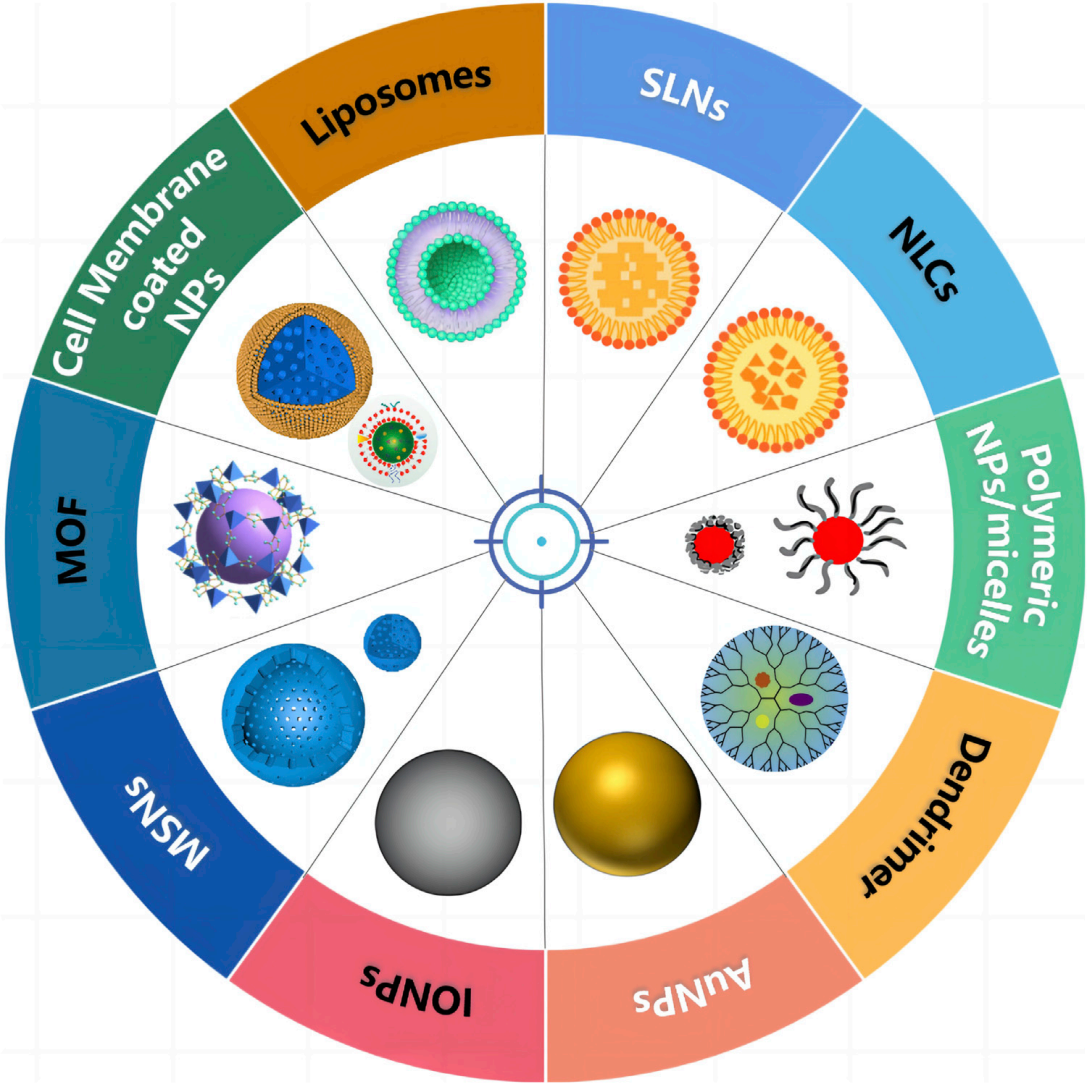
Schematic illustration of the major types of co-delivery systems for cancer therapy.

Various nanoscale carriers such as lipid-based NPs, polymeric NPs, dendrimers, and inorganic systems (magnetic NPs, carbon nanotubes, nanographenes, silica NPs, and so on) have been used as vehicles for co-delivery of therapeutic agents, as shown in Figure 2 and Table 1. As an ideal carrier for co-delivery, they should have the following characteristics: 1) considerable stability, small size, and desired drug loading; 2) loading both hydrophobic and hydrophilic drugs; 3) protecting the loaded drug against degradation; 4) binding with a variety of target molecules with high binding affinity; 5) easy modification; and 6) good biocompatibility.

**TABLE 1:** Different nanoscale carriers for the co-delivery of therapeutic agents in cancer therapy.

Nanocarrier type	Nanostructure	Therapeutics	Cell line	Author (year)
Lipid-based nanocarrier	Liposome	DNR and DIO	A546	Wang et al. (2019)
		Rapamycin (RPM) and regorafenib	CT26	Chen et al. (2020)
		DTX and siPGAM1	A549	Zhang et al. (2021)
		PTX and CBP	SKOV-3	Tang et al. (2022)
	Niosomes	DOX, QC, and siRNA	AGSMCF10A, MCF7, and 4T1	Hemati et al. (2019)
		DOX and d-Limonene	HepG2	Assali et al. (2022)
	NE	DOX and d-Limonene	HepG2	Assali et al. (2022)
		PTX and DHA	MCF-7	Li et al. (2022)
	SLN	PTX and pDNA	MCF-7	Yu et al. (2016)
		PTX and 17-AAG	MKN45	Ma et al. (2018)
		DOX and pEGFP	A549	Han et al. (2014)
	NLC	Erlotinib and RES	A549	Asadollahi et al. (2022)
		IRN and QUE	HT-29	Liu et al. (2022)
Polymeric nanocarrier	Polymeric nanoparticle	miR-21i and DOX	MDA-MB-231 and A549	Sriram and Lee (2021)
	Polymeric micelles	RES and DTX	MCF-7	Guo et al. (2019)
		DOX and IR	LLC and SKOV-3	Wu et al. (2020)
		CDDP and PTX	A2780	Desale et al. (2013)
		PTX and 4-HPR	A2780s	Wang et al. (2020)
	Polymersome	DOX and CQ	MCF-7 and HL60	Xu et al. (2016)
	Polyplex	DOX and siRNA	MCF-7/ADR	Gao et al. (2019)
		DOX and miR-23b	A549	Tang et al. (2021)
Dendrimer	PPI	DO and pTRAIL	MCF-7	Ebrahimian et al. (2018)
	Poly (L-lysine)	CA and DOX	KB	Zhang et al. (2018)
		PLK1 and siRNA	Human MPM	Shi et al. (2021)
	PAMAM	Pt and DOX	MCF-7 and MDA-MB-231	Guo et al. (2019)
		CUR and Bcl-2 siRNA	HeLa	Ghaffari et al. (2020)
		DOX and TRAIL	C26	Pishavar et al. (2019)
Inorganic nanocarrier	AuNP	siRNA and IM	B16F10	Labala et al. (2017)
		GEM and miR-21i	SW1900	Lin et al. (2018)
	IONP	DOX and CDDP	MCF-7	Khafaji et al. (2019)
		PTX and RPM	MCF-7	Dilnawaz et al. (2010)
	MSN	Tax and CUR	7364	Lin et al. (2018)
	MOF	DOX and DZ	A549 and MDA-MB-231	Nie et al. (2020)
		GOx and 1-MT	B16F10	Dai et al. (2022)
		Ce6 and Cyt c	HeLa	Ding et al. (2020)
			SMMC7721	

### 3.1 Lipid-based NPs for co-delivery of therapeutic agents

Lipid-based nanocarriers with features such as high drug loading efficiency, good stability, good biocompatibility, and controllable drug release have been widely used in drug delivery. Lipid-based carriers can be divided into the following categories: liposomes, niosomes, nanoemulsions (NEs), solid lipid NPs (SLNs), and nanostructured lipid carriers (NLCs). On the market or in clinical trials, liposomal or lipid-based products are the most common nanocarriers for drug delivery. Liposomes, one of the most promising drug delivery systems, are usually composed of phosphatidylcholine and cholesterol. Hydrophobic drugs can be encapsulated in the lipid bilayers, while hydrophilic drugs can be easily encapsulated in the aqueous core (Eloy et al., 2014). Liposomes have been widely developed to deliver multiple chemotherapeutic agents in recent years. Among them, two liposomal formulations of small molecular drug combinations are currently being tested in clinical trials. One is CPX-351 (cytarabine (Ara-C)/daunorubicin (DNR)) in patients with acute myeloid leukemia. The other is CPX-1 (irinotecan (IRN)/floxuridine) in patients with colorectal cancer (Weissig et al., 2014). As a nanoscale carrier, liposomes can accumulate at the tumor site due to the enhanced permeability and retention (EPR) effect. In addition to the passive targeting mentioned above, liposomes can also achieve active targeting through their decoration with proteins, antibodies, aptamers, or polypeptides (Qi et al., 2017), such as folate, HA, epidermal growth factor, and transferrin (Qian et al., 2002; Zalba et al., 2016; Zununi et al., 2017; Riaz et al., 2019; Baskararaj et al., 2020; Wang et al., 2020). Wang et al. constructed PFV peptide-modified liposomes for the co-delivery of DNR and dioscin (DIO) to treat non-small cell lung cancer (NSCLC). As a targeting liposomal drug carrier, the cellular uptake of the PFV peptide-modified liposomes by A549 cells was enhanced. The targeted DNR and DIO co-delivery liposomes exhibited significant antitumor effects in tumor-bearing mice (Wang et al., 2019). In another study, dual-targeting liposomes modified with T7 and ^D^A7R were successfully developed to deliver DOX and VCR simultaneously to gliomas. This system could penetrate the blood–brain barrier (BBB) and blood–tumor barrier (BTR). In this study, T7 was a seven-peptide ligand of transferrin receptors (TfRs) with the ability to circumvent the BBB and then target glioma. ^D^A7R, which is overexpressed during angiogenesis, is a D-peptide ligand of VEGF receptor 2 (VEGFR 2) with excellent glioma-homing properties. DOX- and VCR-co-loaded T7/^D^A7R-liposomes showed the most favorable anti-glioma effect *in vivo*. To attain the maximum therapeutic effect and overcome MDR, Liu et al. designed HA-decorated NLCs as nanocarriers for delivering baicalein (BCL) and DOX (HA-BCL/DOX-NLCs). The nanocarriers showed the highest cytotoxicity and synergistic effect of the two drugs in tumor cells (Liu et al., 2016).

### 3.2 Polymeric NPs/micelles for co-delivery of therapeutic agents

Self-assembly polymeric NPs/micelles, characterized by the ability to increase the solubility and stability of anti-tumor agents, excellent biocompatibility, targeting ability, long circulation, and easy production, are very attractive for co-delivery of antitumor agents (Han et al., 2019). Polymeric NPs/micelles, mainly composed of amphiphilic block copolymers, have been applied to simultaneous encapsulation of hydrophilic and hydrophobic anti-tumor agents. Guo et al. designed mPEG-PDLA micelles loaded with DTX and resveratrol (RES) to treat breast cancer. The drug-loaded micelles showed prolonged release profiles and higher cytotoxicity *in vitro* against MCF-7 cells. The AUC_(0→t)_ values of DTX and RES in the micelles after *i. v.* administration to rats were 3.0- and 1.6-fold higher than those of the free DTX and RES. As a result, DTX- and RES-loaded micelles could exhibit improved antitumor effects (Guo et al., 2019). More interestingly, a 2-deoxyglucose (2-DG) prodrug-based micellar carrier, poly-(oligo ethyleneglycol)-co-poly (4-((4-oxo-4-((4-vinylbenzyl) oxy) butyl) disulfaneyl) butanoicacid)-(2-deoxyglucose) (POEG-p-2DG), was also developed as a dual-functional carrier for the delivery of water-insoluble V9302. The micelles exhibited slow-release behavior. V9302 and POEG-p-2DG in combination led to inhibition of the compensatory metabolic shift to glucose metabolism and a more effective inhibition of glutamine uptake owing to POEG-p-2DG-mediated inhibition of ASCT2 glycosylation. The micelles showed higher cytotoxicity toward cancer cells than V9302 alone and significantly improved antitumor activity (Luo et al., 2020). For hydrophobic drugs, a mixed micellar formulation is more attractive for *i. v.* administration. A new TPGS-Soluplus^®^ mixed micelle was constructed for the co-delivery of PTX and fenretinide (4-HPR). The cytotoxicity of the mixed micelles in A2780s cells was reduced by 7.3 and 25.1 times that of free PTX and 4-HPR, respectively. Furthermore, the drug-loaded mixed micelles showed higher AUC and t_1/2_ values than free PTX and 4-HPR, showing better antitumor activity than other groups (Wang et al., 2020). Natural polysaccharides with a high biocompatibility and biodegradability can also self-assemble into polymeric NPs/micelles, such as dextran (DEX). A dextran-based amphiphilic polymer (DEX-DOCA) was designed to deliver PTX and silybin (SB). Moreover, PTX and SB were released from DEX-DOCA at the prospective dosage ratio, specifically in the acid endo/lysosome mimic environment. SB sensitized the cytotoxicity and cell apoptosis induction ability of PTX to A549 cells *in vitro*. Finally, PTX- and SB-co-loaded DEX-DOCA effectively accumulated at tumor sites by the EPR effect and inhibited tumor growth through enhanced intratumoral penetration within the A549 xenograft model (Huo et al., 2020).

### 3.3 Dendrimer for the co-delivery of therapeutic agents

Among the different types of carriers used as co-delivery systems, dendrimers, including peptide dendrimers (PPI), poly (L-lysine) dendrimers, polyamidoamine (PAMAM) dendrimers, and PAMAM-organosilicon dendrimers (PAMAMOS), specifically PAMAM, dendrimers have attracted much attention as nanoscale carriers because of their high positive charge, providing shelter for DNA from nuclease digestion in serum, “proton sponge” effect leading to endosomal escape, controllable size, lipophilicity, monodispersity, unique architecture, highly controlled molecular structure, subcellular size, and biocompatibility (Guo et al., 2019), which makes them good candidates for gene and drug delivery (Abedi-Gaballu et al., 2018; Pishavar et al., 2019; Ghaffari et al., 2020). To improve the solubility, bioavailability, and anti-tumor effect of CUR and Bcl-2 siRNA, PAMAM-CUR/Bcl-2 siRNA NPs were produced. The NPs exhibited more effective cellular uptake and higher inhibition of tumor cell proliferation compared to other formulations. However, direct applications of unmodified PAMAM dendrimers may be limited by their toxicity. To address the problem, various strategies, such as surface functionalization, have been adopted to improve the biocompatibility, transfection efficiency, and cytotoxicity of PAMAM dendrimers. In Pishavar et al. (2019), the primary amine surface of PAMAM (G5) was substituted with cholesteryl chloroformate (5%) and alkyl-PEG (3%) to deliver DOX and TRAIL plasmids, which exhibited much stronger antitumor effects against C26 colon carcinoma cells with lower carrier cytotoxicity(Pishavar et al., 2019).

### 3.4 Inorganic carriers for co-delivery of therapeutic agents

Many inorganic carriers have also been developed for co-delivery of therapeutic agents, such as gold NPs (AuNPs), iron oxide NPs (IONPs), mesoporous silica NPs (MSNs) (Miao et al., 2017), and metal-organic framework (MOF)-based NPs. Labala et al. prepared layer-by-layer assembled AuNPs (LbL-AuNPs) to co-deliver STAT3 siRNA and imatinib mesylate (IM) to treat melanoma. STAT3 siRNA and IM in combination showed greater inhibition of STAT3 protein expression, decreased cell viability, and increased apoptotic activity in B16F10 melanoma cells. In melanoma tumor-bearing mice, LbL-AuNP showed a significant (*p* < 0.05) reduction in tumor volume, tumor weight, and STAT3 protein expression compared with control treatments (Labala et al., 2017). As for IONPs, citric acid-functionalized Fe_3_O_4_ magnetic NPs (CA-MNPs) were employed as carriers to deliver DOX and melittin (MEL) for the treatment of cancer. The results of *in vitro* cytotoxicity showed that DOX- and MEL-loaded CA-MNPs had a synergistic effect in inhibiting MCF-7 cell growth, substantially leading to greater anti-tumor efficacy, compared to DOX or MEL alone (Hematyar et al., 2018). The advantages of using AuNPs and IONPs as carriers for the co-delivery of therapeutic agents were their tunable size and shape and facile tracking by X-ray computed tomography imaging and magnetic resonance imaging, respectively. However, the clinical application of AuNPs and IONPs is limited because of their instability, low drug-loading efficiency, and potential toxicity. Compared to AuNPs and IONPs, drugs with various physicochemical properties can be loaded simultaneously into MSNs with high loading efficiency (Dilnawaz et al., 2010; Ashley et al., 2011; Xiao et al., 2012; Mieszawska et al., 2013). Meng et al. prepared functionalized MSNs to deliver P-gp siRNA and DOX into the drug-resistant human cervical cancer cell KB-V1. The dual delivery system could increase the intracellular as well as intranuclear drug concentration in KB-V1 cells, thus improving the drug sensitivity of KB-V1 cells to DOX (Meng et al., 2010). MOF-based NPs with high loading capacity and good enzyme fidelity have been identified as ideal vehicles. A pH/ROS dual-responsive degradable MOF nanoreactor-based nanosystem loaded with GOx and 1-MT has been designed with self-amplified release and enhanced penetration for the combined starvation/oxidation therapy and IDO-blockade immunotherapy of tumors. The co-delivery system rapidly disassembled in response to the intracellular reactive oxygen species (ROS) in tumor cells, thus releasing GOx and 1-MT. GOx competitively consumed glucose and generated ROS, further inducing self-amplifiable MOF disassembly and drug release. The starvation/oxidation combined IDO-blockade immunotherapy not only strengthened the immune response and stimulated the immune memory through the GOx-activated tumor starvation and recruitment of effector T cells but also effectively relieved the immune tolerance by IDO blocking, remarkably inhibiting tumor growth and metastasis *in vivo*.

The exploration of nanoscale carriers for the co-delivery of therapeutic agents has made significant strides in advancing drug delivery systems tailored to the intricacies of various diseases, particularly cancer. The selection and customization of these nanocarriers are critical, hinging on their ability to encapsulate and protect both hydrophobic and hydrophilic drugs, achieve targeted delivery, and respond to the unique microenvironment of tumors or disease sites. Lipid-based nanoparticles (NPs), such as liposomes and nanostructured lipid carriers, have emerged as frontrunners in the field, prized for their high drug loading efficiency, biocompatibility, and versatility to accommodate different drug types. These carriers exploit the EPR effect for tumor targeting and can be further modified for active targeting using ligands that bind specifically to receptors overexpressed on tumor cells. This dual targeting approach, leveraging both passive (EPR) and active mechanisms, significantly improves the delivery of therapeutic agents directly to the tumor site, minimizing systemic side effects and enhancing therapeutic efficacy. Polymeric NPs and micelles, characterized by their self-assembling properties and ability to solubilize both hydrophobic and hydrophilic drugs, offer another promising avenue. They can be engineered for prolonged circulation, targeted delivery, and controlled release of drugs, responding to specific triggers within the tumor microenvironment, such as pH or enzyme presence. This adaptability makes them particularly suitable for delivering combinations of drugs designed to work synergistically for maximum therapeutic impact. Dendrimers and inorganic carriers like AuNPs and IONPs present unique benefits, including high stability, tunable size, and functionalizability for targeted drug delivery. Dendrimers, for example, offer a highly controlled structure for delivering genes and drugs, with modifications to enhance biocompatibility and reduce toxicity. Inorganic NPs, while facing challenges related to stability and toxicity, hold potential for multifunctional applications, including imaging and therapy, owing to their physical properties. The choice of the carrier is influenced not only by the physicochemical properties of the therapeutic agents but also by the specific requirements of the target site, such as the need to cross biological barriers or target specific cell types within the tumor microenvironment. Moreover, the design of these nanocarriers often considers the need for environmental responsiveness, ensuring that the release of the encapsulated drugs is precisely controlled, enhancing efficacy while reducing potential side effects. In summary, the selection and design of nanoscale carriers for co-delivery systems are a delicate balance of the carrier’s inherent properties, the specific demands of the therapeutic agents, and the disease microenvironment. This approach underscores a move toward more personalized and precise medical treatments, where the choice of delivery systems is as crucial as the choice of drugs in achieving optimal therapeutic outcomes.

## 4 Drug-loading strategies of co-delivery systems

To meet the requirements of different drug release behaviors and anti-tumor effects, different drug-loading strategies have been developed to effectively encapsulate different therapeutic agents, ensuring optimized drug loading and controlled release. The strategies include simple simultaneous loading of drugs, a prodrug as a carrier for simultaneous loading drugs, and a drug as a carrier for simultaneous loading drugs.

### 4.1 Simple simultaneous loading of drugs

Among different drug-loading strategies, simple simultaneous loading of drugs is the most common strategy for co-delivery. Yang et al. developed a novel lipid NP loading DOX and CUR simultaneously for liver cancer treatment. The novel lipid NPs were formulated with a high-pressure microfluidics technique. In brief, the lipid phase was dissolved in ethanol and heated to 75°C. DOX and CUR were then added to the lipid sample. After the removal of the solvent by rotary evaporation, preheated water was added gradually to the hot and molten lipid sample and gently magnetically stirred. A coarse oil-in-water emulsion was formed by high-speed shearing via a homogenizer. The coarse emulsion was further homogenized for six cycles at 1,000 bar with a high-pressure microfluidics device. The hot dispersion was cooled down to 4°C and sterilized using a 0.45-μm filter (Zhao et al., 2015). Simple simultaneous loading of drugs is also suitable for simultaneously loading both hydrophilic and lipophilic drugs. Jing et al. constructed epirubicin (EPB)- and schisandrin B (SSD B)-loaded PFV-modified liposomes. In these liposomes, lipophilic SSD B was encapsulated in the phospholipid bilayer as a potential inhibitor for tumor metastasis. Hydrophilic EPB was used as an antitumor agent and was entrapped into the hydrophilic inner core of the liposomes. Or even more subtly, Meng et al. prepared a lipid-coated mesoporous silica nanoparticle platform for synergistic GEM and PTX delivery to human pancreatic cancer cells in mice. GEM was loaded into the MSN core and hydrophobic PTX was incorporated into the lipid bilayer (Meng et al., 2015).

### 4.2 Prodrug as a carrier for simultaneous loading of drugs

A prodrug as a carrier for co-delivery is another strategy for drug loading. The prodrug carrier usually consists of three parts: polymer, truss arm, and drug. The prodrug carrier usually starts with a chemical reaction or polymer polymerization, which means linking the polymer chain segment with the truss arm and then with the drug. The hydrophobic and hydrophilic properties of the prodrug carrier can be self-assembled after bonding with drugs, which will integrate prodrug and nanodrug delivery technology into a more efficient drug loading method. For example, a 2-DG prodrug-based micellar carrier (POEG-p-2DG) well that retained the pharmacological activity of 2-DG was developed. Moreover, POEG-p-2DG could self-assemble to form micelles and load V9302. The micelles exhibited slow-release kinetics *in vitro*, showed more cytotoxicity toward cancer cells than V9302 alone, and significantly improved anti-tumor activity (Luo et al., 2020). In another research, prodrug-based micellar carrier poly (DL-lactide-co-glycolide)-b-poly (ethylene glycol)-carboplatin (PLGA-PEG-CBP) was synthesized to co-encapsulate PTX and CBP. The obtained PTX/CBP NPs showed strong cytotoxicity toward tumor cells *in vitro* and significantly inhibited tumor growth *in vivo* (Zhang et al., 2016). When designing prodrugs, we should consider the characteristics of the drugs to choose the appropriate polymer and truss arm. When the drug is linked to a polymer carrier, impurities may be mixed in the target products. Therefore, it is necessary to strengthen the quality control of the active drug and carrier before linking and reaction process control.

### 4.3 Drug as a carrier for simultaneous loading of drugs

The conventional carriers for co-delivery have low drug-loading capacity, thus reducing the therapeutic effect (Mohammad et al., 2018b). Drugs as carriers for co-delivery are also another strategy for drug loading. Drugs used as carriers serve not only as adjuvants but also exert their therapeutic effects. Rubusoside (RUB), a natural steviol glycoside, has loaded several lipophilic compounds, including PTX, CUR, and etoposide, for *in vivo* studies (Jeansonne et al., 2011; Zhang et al., 2011; Zhang et al., 2012; Liu et al., 2015). Liu et al. (2015) prepared PTX-RUB NPs to improve the solubility of PTX. RUB could self-assemble to form micelles and load PTX. In addition to addressing the solubility issue of PTX, RUB, as a solubilizer and a P-gp inhibitor, was also capable of overcoming cell permeability barriers (Liu et al., 2015). Mohammad et al. (2018b); Mohammad et al. (2019) also designed a drug-delivering-drug (DDD) platform based on the PTX-disulfiram nanococrystals, which possessed a high drug-loading capacity at a precise co-drug ratio for MDR reversal and enhanced anti-tumor effects *in vivo* and *in vitro* (Mohammad et al., 2018b; Mohammad et al., 2019). Another drug, glycyrrhizic acid (GA), one of the main active ingredients of traditional Chinese medicine, *Glycyrrhiza glabra L*, has good anti-inflammatory, antiviral, antitumor, and other biological activities. It has been adopted to improve the solubility of insoluble drugs because of its amphiphilic characteristics (Yang et al., 2015). When the concentration of GA reaches the critical micelle concentration (CMC), the non-covalent bond drives the aglycone structure to form a hydrophobic core and sugar chains to form hydrophilic shells, and then GA self-assembles to form micelles and effectively increases the solubility of insoluble drugs. For example, andrographolide (AP)-loaded GA micelles were prepared to enhance the solubility and anti-tumor effect of AP. The micelles were prepared using the thin film dispersion method: AP and GA were added and mixed with ethanol. The organic solvent was then removed by rotary evaporation at 37°C under reduced pressure to form a thin film. Next, the obtained film was hydrated for 4 h. After centrifugation for 10 min, AP-loaded micelles were in the clear and transparent supernatant. In this research, using GA as a carrier could not only improve the solubility of AP but also have a synergistic effect on AP to improve the anti-tumor effect (Li et al., 2018).

The synergistic effects of combined therapy are highly dependent on the relative concentration of the drug combinations in tumor tissues. How to achieve the preset drug loading and the constant ratio of multiple drugs in the co-delivery system and stably deliver them into tumor tissues claims our highest attention. This requires us to choose the appropriate carrier and drug loading strategy according to the drug properties, drug release behaviors, and anti-tumor effect. Generally, if the drug is loaded with the same strategy or the release of the drug is controlled in the same way, the release rates of the multiple drugs are relatively synchronous. For example, if both drugs are physically wrapped in micelles at the same time, the release rates of the two drugs are relatively synchronous. When the drug is loaded in different ways, such as when two drugs are physically wrapped and chemically linked, respectively, different release environments will inevitably result in an unsynchronized release sequence and rate of the drug.

## 5 Nanoscale-controlled/targeted co-delivery systems

**FIGURE 3:**
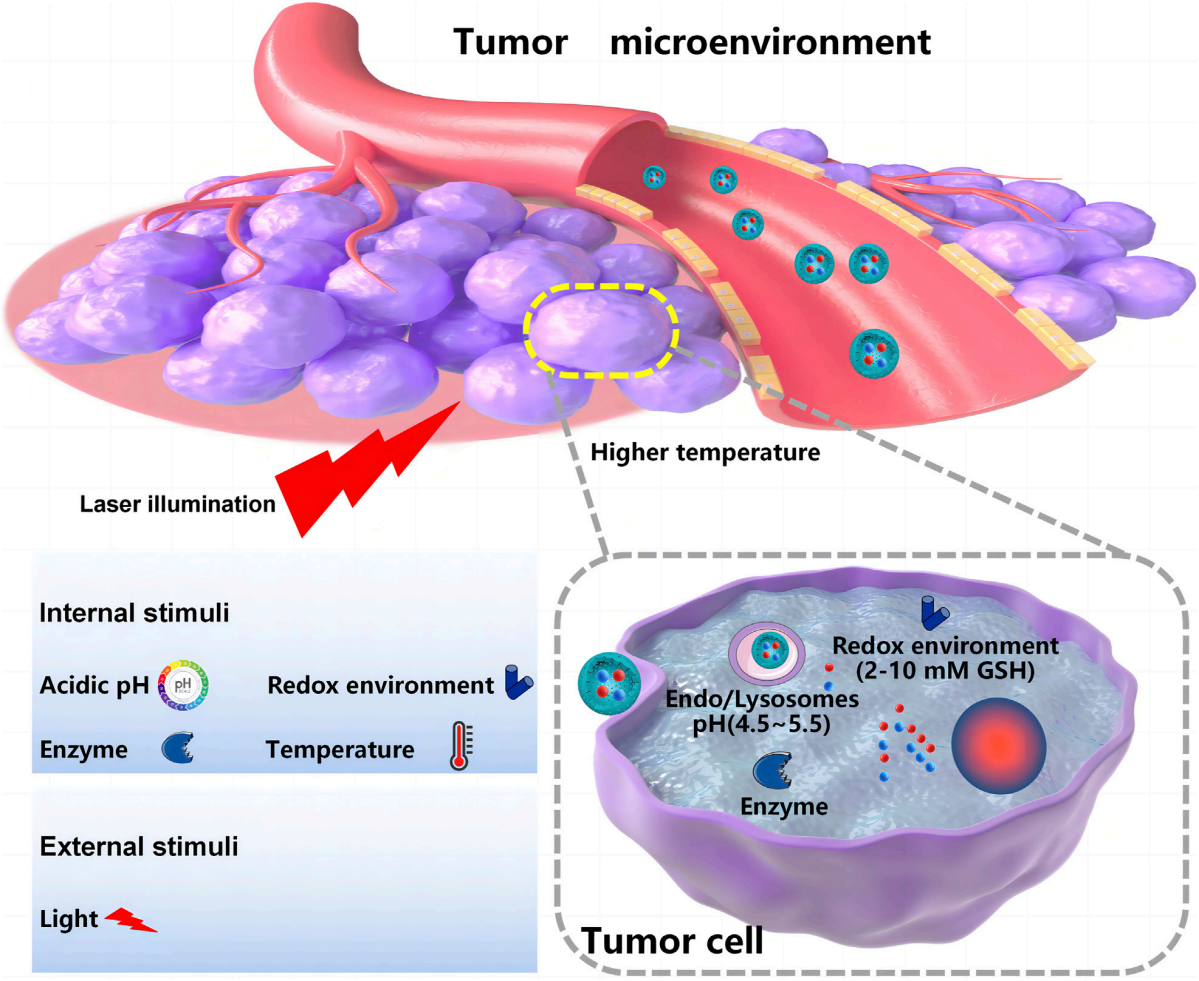
Nanoscale-controlled co-delivery systems.

**FIGURE 4:**
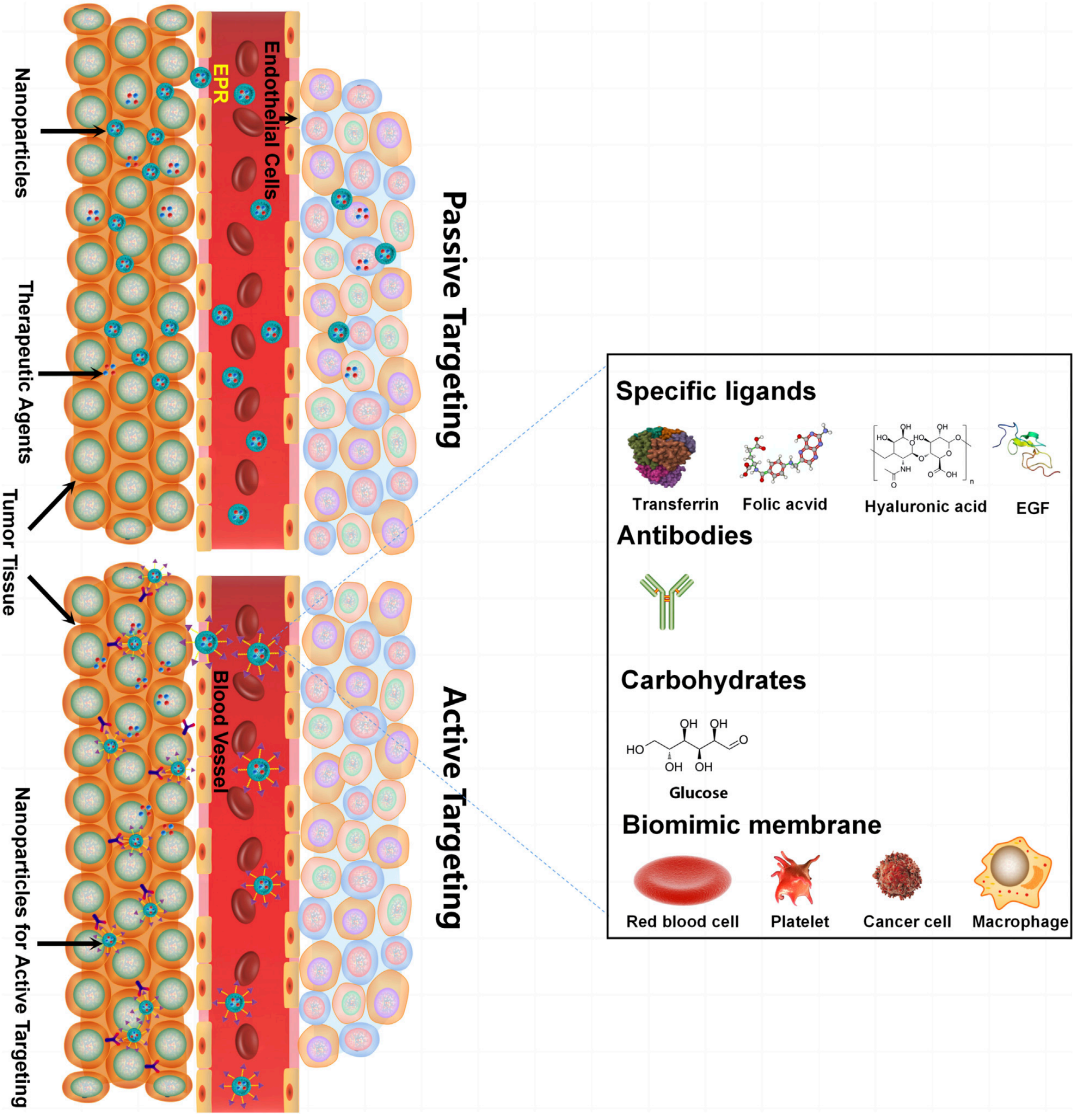
Nanoscale-targeted co-delivery systems.

Controlled release and delivering specific drugs to the tumor site remain the main challenges for co-delivery systems developed for oncotherapy. Compared with the normal human internal environment, the tumor microenvironment has unique features. These features can be pH- or redox potential-related or enzyme-, temperature-, or receptor-mediated (Balaure et al., 2018), which provides chances for internal triggering and targeted co-delivery systems. The external triggering co-delivery systems are also designed to respond to outside intervention, such as light or magnets. As shown in Figures 3, 4, nanoscale-controlled/targeted co-delivery systems have been widely applied in cancer treatment.

### 5.1 Nanoscale-controlled co-delivery systems

#### 5.1.1 pH-responsive co-delivery systems

Because of an insufficient supply of oxygen and nutrients, tumor tissue has a more acidic environment (pH 6.0–7.2) than normal tissue or blood (pH 7.4) (Neumann et al., 2017). The pH values will decrease even further in intracellular organelles, such as endosomes (5.0–6.0) and lysosomes (pH 4.5–5.0) (Ghaffari et al., 2018; Ahmadi et al., 2022). Because the pH differences in the body are relatively simple and intrinsic, pH-sensitive NPs are frequently employed in delivering therapeutic agents for the treatment of cancer (Yang et al., 2017). pH-triggered protonation/ionization is widely applied in the preparation of pH-responsive NPs. Certain ionizable groups, such as amino, carboxyl, sulfonate, pyridine, and imidazolyl groups, have been introduced into the structure of NPs to realize pH-responsive delivery (Alsawaftah et al., 2022). Exposure to low pH leads to protonation or charge reversal of the introduced ionizable group. Consequently, the hydrophilic–hydrophobic balance inside the NPs is broken, and the nanocarrier’s structure is disassembled, thus releasing the encapsulated drugs (Alsawaftah et al., 2022). A pH-responsive SRF and anti-miRNA27a co-loaded anti-GPC3 antibody targeting lipid NPs was designed by Wang et al. to increase the therapeutic efficacy against liver cancers. The protonation of the central pyridine structure of cationic switchable lipid in the lipid NPs allowed the switching toward intramolecular hydrogen bonding at lower pH in the tumor, thereby modifying the hydrocarbon chain orientation and leading to the release of SRF and anti-miRNA27a after nanoparticle disassembly. The co-delivery of SRF and anti-miRNA27a showed a marked increase in the proportion of apoptosis tumor cells than that of free SRF. *In vivo* animal studies in the liver cancer xenograft model witnessed significant suppression of tumor growth with elevated TUNEL-positive apoptosis after administration of the pH-responsive lipid NPs. In addition, pH-sensitive chemical bonds, such as imine, hydrazone, oxime, amide, ethers, and orthoesters, are used to construct pH-responsive NPs. The pH-sensitive NPs are stable under physiological conditions, but in the acidic tumor environment, the chemical bonds can be broken, releasing the encapsulated payload (Yan and Ding, 2020). Tao et al. developed a pH-responsive nanoparticle carrier based on pectin (PET). The platform loading DOX and DHA released DOX at the tumor site due to the weakly acidic tumor environment by breaking the acid-sensitive acyl hydrazone bond of the NPs. The cellular uptake confirmed that these PET-DOX/DHA NPs efficiently delivered DOX into the nuclei of MCF-7 cells. The PET-DOX/DHA NPs significantly inhibited tumor growth in tumor-bearing mice (Tao et al., 2021). Moreover, some pH-responsive NPs, MOFs, for example, are designed with specific bond arrangements that are sensitive to environmental pH. MOFs have been widely investigated as pH-responsive NPs in recent years. Duan et al. constructed pH-responsive MOF-based NPs to deliver immunostimulatory unmethylated cytosine phosphate–guanine oligonucleotide (CpG) and tumor-associated antigens (TAAs) for cancer immunotherapy. Antigen release reached approximately 60% under pH 5.0 because the coordination of Eu and guanine monophosphate (GMP) dissociated under pH 5.0, which facilitated protein vaccine escape from the endo/lysosome (pH 4.5) into the cytosol (Duan et al., 2017; Alsawaftah et al., 2022).

In recent years, great progress has been made in site-specific drug release by studying the tumor microenvironment and using its unique pH response factors. However, most of the research studies on pH-responsive co-delivery systems are still in the experimental stage, and there are still many difficulties to be solved, such as the biocompatibility and toxicity of the carriers. With the rapid development of nanotechnology and polymer materials, it is believed that pH-responsive nanocarriers will play a great role as drug carriers in the near future.

#### 5.1.2 Redox-responsive co-delivery systems

Among different internal stimuli-responsive systems, redox-responsive drug delivery systems are considered efficient because of the GSH concentration difference between tumor and normal cells. In tumor tissues, intracellular and extracellular environments exhibit high redox potential differences, with GSH concentrations in tumor cells approximately 7–10 times higher than those of normal cells, which provides a unique opportunity to use redox-responsive delivery systems for cancer therapy. Redox-responsive agents, such as disulfide bonds (-S-S-) and diselenide bonds (-Se-Se-), are frequently used in redox-responsive drug delivery systems. There are two ways to introduce redox-sensitive bonds: one is to directly insert redox-sensitive bonds into the nanocarrier and the other is to use precursor drugs containing redox-sensitive bonds chemically conjugated onto NPs (Mollazadeh et al., 2021). The redox-responsive nanocarriers are often stable in normal tissues and show a prompt response to high GSH concentration in tumor cells to release therapeutic agents. The disulfide bond has been proved to be the most widely used redox-responsive bond to develop redox-responsive drug co-delivery systems. For direct introduction of disulfide bonds, Zhao et al. constructed redox-responsive MSNs loading DOX/Bcl-2 siRNAs for targeted intracellular drug release and synergistic therapy, in which Bcl-2 siRNAs were connected to the MSN surface via disulfide linkers and acted as GSH-sensitive gatekeepers of mesopores (MSNs-SS-siRNA), avoiding the release of siRNA/DOX into blood after injection. After uptake into tumor cells and exposure to intracellular GSH levels, the disulfide linkages in the NPs were disrupted, which led to the release of siRNA/DOX (Zhao et al., 2017). Apart from the direct introduction of disulfide bonds, disulfide bonds can also be indirectly inserted into nanocarriers in the form of precursor drugs. For instance, Sauraj et al. developed a CUR/5-FU co-delivery system for cancer treatment. The Xyl-SS-CUR conjugate synthesized via covalent conjugation of CUR to xylan through an -S-S- linkage could self-assemble into NPs, and the lipophilic 5-fluorouracil-stearic acid (5-FUSA) prodrug was encapsulated into the hydrophobic core of Xyl-SS-CUR NPs. The Xyl-SS-CUR/5-FUSA NPs showed excellent redox-responsiveness owing to the disulfide linkage, releasing both CUR and 5-FUSA in the presence of GSH (Sauraj et al., 2020). Of the two methods, the indirect method is simpler and more commonly employed in the development of redox-responsive drug co-delivery systems.

However, there are still some problems existing in redox-sensitive drug delivery systems: 1) it cannot achieve tumor targeting alone. Redox-sensitive drug delivery systems currently need to be combined with other target modifications to realize tumor targeting. 2) The complexity of its structural design still needs to be improved: drug delivery systems often need layer-by-layer modification to achieve its targeting while introducing redox-sensitive bonds, which increases its structural complexity and is not conducive to preparation and stability. 3) The *in vivo* kinetics after redox reactions need to be studied further. Nonetheless, the rational design of new redox-sensitive nanomaterials and a detailed understanding of their *in vivo* kinetics is undoubtedly the trend of developing novel redox-sensitive drug delivery systems.

#### 5.1.3 Enzyme-responsive co-delivery systems

Enzyme-responsive NPs have been considered one of the most promising smart stimulus-responsive NPs (Li et al., 2020). Concentration gradients of specific enzymes, such as matrix metalloproteinases (MMPs), hyaluronidase (HAase), cathepsin B, and esterase, between tumor tissues and normal tissues (usually tumor tissue > normal tissue) can be used as appropriate activators for achieving localized drug release (Jhaveri et al., 2014; Ghaffari et al., 2018; Rahikkala et al., 2018). At present, among different enzyme-responsive nanocarriers, MMP-responsive nanocarriers have been widely studied. Tan et al. constructed an enzyme-responsive polymeric nano-drug delivery system (NPs@Ce6) for the treatment of bladder cancer. The enzyme-responsive function was realized with an enzyme-responsive diblock poly (OEGMA)-PTX prodrug, and Ce6 was encapsulated into it. In the study, PTX or Ce6 could be released rapidly in a buffer containing papain, with the cumulative release percent at 80% within 8 h. In contrast, without papain, the cumulative release percent of PTX or Ce6 was less than 20% in 24 h, indicating the enzyme-responsiveness of NPs@Ce6 (Tan et al., 2021). Considering the overexpression of both the CD44 receptor and HAase in the tumors, He et al. designed cascade-targeting, dual drug-loaded, core-shell NPs (DLTPT) consisting of CD44-targeting HA shells decorated with DOX (HA-DOX) and mitochondria-targeting triphenylphosphonium derivative nanoparticle cores loaded with lonidamine (LND) dimers (LTPT). DLTPT showed prolonged blood circulation time and efficiently accumulated at the tumor site owing to the tumor-homing effect and negatively charged HA. Subsequently, the HA-DOX shell was degraded by extracellular hyaluronidase, resulting in decreased particle size and negative-to-positive charge reversal, which would increase tumor penetration and internalization. The degradation of HA-DOX further accelerated the release of DOX and exposed the positively charged LTPT core for rapid endosomal escape and mitochondria-targeted delivery of LND. Notably, when DLTPT was used in combination with anti-PD-L1, tumor growth was inhibited, which induced an immune response against tumor metastasis (He et al., 2021).

The enzyme-responsive diagnosis and treatment system has good sensitivity and specificity, but the types and levels of overexpressed enzymes are quite different in different tumors or different stages of the same tumor. There are also different subtypes of the same enzyme, and the substrates of the same family of enzymes are also similar. Therefore, it is necessary to discover better tumor-specific enzymes and design more ingenious responsive strategies to improve the accuracy and sensitivity of enzyme-responsive treatment systems. The design of enzyme-responsive nanomedicine should pay attention to the kinetics of the enzyme-catalyzed reaction. The enzyme reaction time and reaction degree both affect the drug release rate and therapeutic effect, and the dosage can be optimized according to the enzyme catalytic kinetics.

#### 5.1.4 Temperature-responsive co-delivery systems

Pathophysiological conditions like tumors and infections occur naturally at elevated temperatures. This property can be used as an appropriate strategy to achieve controlled drug release (Rahikkala et al., 2018). Shin DH et al. prepared a temperature-sensitive PEGylated immunoliposome (TSL) with Herceptin-attached encapsulating GCT (Her-PEG-TSL-GCT). The phospholipid dipalmitoylphosphatidylcholine (DPPC) with a transition temperature above 37°C gave the TSL the capacity of temperature-responsiveness. The TSL showed more than 80% drug release within 30 min at 39.4°C. After entering the cancer cells, the TSL could release the drugs due to the high temperature in cancer cells (Shin et al., 2016). In addition to the above-mentioned endogenous stimulus, exogenous heating sources such as electromagnetic, laser, water bath, or even high-intensity focused ultrasound are used to produce a clear temperature contrast between normal and tumor tissues. A thermosensitive liposome-coated poly (N-isopropylacrylamide-co-acrylamide) (P(NIPAM-co-AAM)) nanogel loaded with ICG and DOX (DI-NGs@lipo) was prepared. The core of the nanogels made the DI-NGs@lipo possess a volume-phase transition temperature (VPTT) near 40°C. Under NIR light irradiation, the DI-NGs@lipo with a significant photothermal effect triggered the release of DOX, showing the chemo-photothermal synergistic therapeutic effects on tumor cells (Yu et al., 2018).

Temperature-responsive co-delivery systems are considered significantly important besides other stimulus-sensitive factors such as pH, light, and enzymes. On one hand, the temperature of tumors is higher in comparison with body temperature and can act as an endogenous stimulus in order to activate nanocarriers, thus releasing the therapeutic cargo. On the other hand, external heating sources such as electromagnetic, laser, water bath, or even high-intensity focused ultrasound at an exogenous temperature can also activate nanocarrier operation. These provide more possibilities for the use of temperature-responsive co-delivery systems.

#### 5.1.5 Light-responsive co-delivery systems

Light-responsive delivery systems can provide on-demand release capabilities to activate therapeutic effects in a highly spatiotemporal manner. Activated light is categorized by wavelength: ultraviolet light (UV, 100–400 nm), visible light (400–750 nm), and near-infrared light (NIR, 750–2000 nm). Through light irradiation, the release of drugs or the “on–off” control effect can be achieved. Different mechanisms have been suggested to be employed for light-activated drug delivery/release systems, including bond-cleavage, isomerization, cross-linking, electrostatic assembly/disassembly, reduction, oxidation, photocaging/uncaging, as well as nonlinear photoconversion mechanisms such as two-photon absorption and upconversion photoexcitation (Karimi et al., 2017). Zhang et al. (2016) prepared an ICG-ferlactone liposome, which achieved drug release of 96.74% within 8 h of near-infrared light (NIR) irradiation at 808 nm, achieving the effect of on-demand drug release (Zhang et al., 2016). Wu et al. (2018) prepared a photo-responsive mesoporous silica nanoparticle (PMSN) as a co-carrier of the P-GPshrNA and DOX photocage prodrug and achieved orthogonal and continuous release of shRNA and DOX through 405 nm and 365 nm external light, enhancing drug retention and significantly improving the efficacy of chemotherapy against MDR(Wu et al., 2018).

Compared with other intracellular or external stimuli, the light trigger is superior because of its easy manipulation, precisely providing both spatial and temporal control of drug release. However, currently, many of the light-responsive delivery systems require expensive lasers and complex setups of the instruments, which limits their wide use in light-responsive delivery systems. In the future, equipment with simple operation for patients and physicians needs to be produced.

#### 5.1.6 Multiple-responsive-controlled co-delivery systems

Multiple responses to the common stimulus, combined with advantages, make up for the shortcomings of each response and achieve synergistic control of drug release or multiple ways of drug release. Lin et al. fabricated a pH and glutathione (GSH) dual-sensitive co-delivery system loading DTX and rubone (RBN) for the treatment of taxane-resistant (TXR) prostate cancer. After uptake by tumor cells, the micelles underwent expansion and disassembly owing to the protonation of DIPAE and GSH-induced cleavage of -S-S- in acidic tumor cells, releasing DTX and RBN rapidly (Lin et al., 2019). More complicatedly, Wang et al. prepared a pH-responsive DNA and disulfide-linked polyethylenimine-functionalized gold nanorod for specific co-delivery of DOX and pyronaridine (PND) to overcome MDR in the treatment of cancer. The loaded drugs were responsively released from the nanocomplex under acidic pH ∼5, intracellular GSH concentration content at 5 mM, and/or 808-nm NIR laser irradiation, effectively reversing MDR in MCF-7/ADR cells (Wang et al., 2017).

For the future of anticancer co-delivery systems, multiple-responsive carriers, such as pH/temperature, redox/enzyme, pH/redox, and pH/temperature/magnetic, will become the main research hotspot. The multiple-responsive controlled co-delivery systems can exert synergistic effects of different environmentally responsive substances and then overcome the drawbacks of therapeutic agents in delivery. However, the more complicated the design and synthesis of multiple-responsive carriers, the more difficult the quality control becomes.

### 5.2 Nanoscale-targeted co-delivery systems

Delivering synergetic therapeutic agents to their right site at the right dose is also vital for co-delivery systems. Nanomedicine can provide targeted delivery of therapeutic agents selectively to cancer cells in an active targeting or passive targeting way, achieving a cytotoxic concentration higher in tumors with reduced toxicity when compared with free drugs. “Passive targeting” mainly refers to NPs accumulating within the perivascular tumor due to the leaky tumor vasculature and environment, which is called the EPR effect. “Active targeting” mainly refers to NPs linked with tumor-targeting moieties like specific ligands (adenosine receptors, transferrin receptors (TfR), EGFR, folate receptors (FR), integrins, chlorotoxin, somatostatin receptors, and cytokeratin), antibodies (vascular cell adhesion molecule-1 (VCAM-1) antibody and anti-EGFR antibody), and carbohydrates (glucose, mannan, and cellulose) for cancer targeting (Table 2) (Ahmad et al., 2019). For specific ligand-mediated “active targeting,” the folate-conjugated co-delivery systems are very common with compounds including chemotherapeutic agents, small-molecule inhibitors, silencing RNA, miRNAs, chemokines, and combinations of therapeutic agents. Chen et al. designed a new folate-decorated amphiphilic bifunctional pullulan-based copolymer (FPDP) to deliver DOX and short hairpin RNA of Beclin1 (shBeclin1). The micelles loaded with DOX and shBeclin1 enhanced the anti-tumor effect of DOX by blocking the Beclin1 protein-mediated autophagy process, leading to synergistic cell apoptotic induction in HeLa cells and revealing superior anti-tumor efficacy of FR-targeted micelles when compared with non-FR-targeted micelles or free DOX (Chen et al., 2018). For antibody-mediated “active targeting,” Yalamarty et al. (2022) prepared 2C5 antibody-targeted (mAb 2C5) dendrimer-based mixed micelles for co-delivery of siRNA and DOX (Yalamarty et al., 2022). The tumor-specific mAb 2C5 attached to the micelles led to improved tumor targeting and increased accumulation of siRNA and DOX at the tumor site, thus showing a better anti-tumor effect. Interestingly, carbohydrates can also act as tumor-targeting moieties. For example, glucose-coated NPs showed differential uptake in metabolically active normal cells and cancer cells due to the overexpression of the glucose transporter (GLUT) in cancer cells. Wang et al. fabricated novel glucose-functionalized polymeric micelle NPs for the co-delivery of Ara-C and fluorodeoxyuridine (FUDR). HepG2 cells showed enhanced cellular uptake of the glucose-functionalized NPs compared with the non-glucose-functionalized NPs. Therefore, the glucose-functionalized NPs showed better anti-tumor effects *in vitro* than the non-glucose-functionalized NPs or free Ara-C and FUDR. However, most of the traditional NPs mentioned above are likely to be identified and eliminated as foreign substances by the immune system. To address this issue, novel biomimetic NPs have drawn great attention. These biomimetic NPs, taking advantage of the natural properties of many membranes, can act like a “Trojan horse” to evade the immune system. In recent years, cell membrane-camouflaged NPs for active delivery have provided a new strategy for tumor-targeted drug delivery. Red blood cell (RBC) membrane (or erythrocyte membrane), platelet membrane, cancer cell membrane, immune cell membrane, and so on have been coated on NPs to fabricate biomimetic NPs for targeted cancer therapy (Jin et al., 2018; Chen et al., 2019; Chen et al., 2021; Wang et al., 2020). The biomimetic NPs can undergo prolonged circulation to reduce clearance by the immune system and realize homologous targeting toward cancer cells. For example, Yao et al. prepared a cancer-cell-biomimetic NP for the co-delivery of a chemotherapeutic drug (RA-V) and a PD-1/PD-L1 blockade inhibitor (BMS-202). The cancer cell membrane coated with the NPs endows the biomimetic drug delivery system with increased blood circulation and homologous targeting (Yao et al., 2022). Single membrane-coated NPs have been extensively studied due to their ability to improve the anti-tumor effect. However, multifunctional fused membrane materials from different membrane types are still rare (Bu et al., 2019), which suggests the potential for preparing NPs coated with different membranes to further improve the anti-tumor effect. For instance, we can simultaneously achieve long circulation with the immune evasion ability of platelet membranes due to surface markers comprising “do not eat me” proteins and tumor targeting with the homotypic targeting capability of cancer cell membranes via specific surface adhesion molecules for co-delivery. Similarly, RBCm and cancer membranes simultaneously fused into one nanoparticle can also achieve long circulation and tumor targeting for co-delivery. Leukocytes combined with cancer membranes and stem cells combined with cancer membranes are also worthy of application for co-delivery. The advantages of cell membrane-camouflaged NPs in tumor therapy have been validated in various studies. In most cases, the biomimetic drug delivery system can perform the functions of their membrane-derived cells, but the following problems need to be solved: 1) the separation and purification protocols of the cell membrane still need to be adjusted and simplified. 2) How to preserve the properties of the membrane during the process of nanopreparation is a matter of concern. 3) The complex transport mechanism of different cell membranes as carriers *in vivo* has not been fully understood and needs further study. 4) For cancer cell membrane-camouflaged NPs, it is noteworthy that those NPs may cause cancer metastasis if the genetic material in the cell is not completely removed.

**TABLE 2:** Active targeting co-delivery systems of therapeutic agents in cancer therapy.

Type of targeting moieties	Targeting moieties	Nanotechnology platform	Therapeutics	Type of study	Cell line	Reference
Specific ligand	Folate	Polymeric micelles	shMCL-1 and PTX	*In vitro* and *in vivo*	Skov3 and A2780T	Nie et al. (2021)
		Cyclodextrin-based nanoformulation	Ginsenoside Rg3 and QUE	*In vitro* and *in vivo*	CT26 and HCT116	Sun et al. (2022)
		Polymeric micelles	2-DG and α-tocopheryl succinate	*In vitro* and *in vivo*	HT29, HeLa, and A549	Lei et al. (2017)
		Micelles	Temozolomide and EGFR-siRNA	*In vitro* and *in vivo*	U87	Wang et al. (2022)
		Hyperbranched polymers	DOX and siRNA	*In vitro*	MDA-MB-468	Zhao et al. (2022)
	Transferrin	SLN	DTX and BCL	*In vitro* and *in vivo*	A549	Li et al. (2017)
		Microemulsion	β-elemene and celastrol	*In vitro* and *in vivo*	A549	Zhang et al. (2019)
Antibody	mAb 2C5	Dendrimer micelles	siRNA and DOX	*In vitro*	MDA-MB-231 ADR and SKOV-3 TR	Yalamarty et al. (2022)
	Anti-GD2 antibody	Liposomes	CPT-11 and panobinostat	*In vitro* and *in vivo*	U87 DR	Jose et al. (2020)
	PSMA antibody (PSMAab)	Bovine serum albumin-polyethylenimine layer-by-layer (LBL) NPs	DTX and siRNA	*In vitro* and *in vivo*	CWR22R	Pang et al. (2017)
Carbohydrates	Glucose	Polymeric micelles	PTX and siRNA	*In vitro*	MCF-7, BT-474	Nasab et al. (2021)
		Polymeric micelles	Ara-C and fluorodeoxyuridine	*In vitro*	HepG2	Wang et al. (2013)
	Mannan	Polymeric micelles	Tumor cell lysate and poly riboinosinic polycytidylic acid	*In vitro* and *in vivo*	4T1	Sheikhzadeh et al. (2021)
Biomimetic cell membrane	Erythrocyte membrane	Erythrocyte membrane-camouflaged polymeric micelles	IR780 and DTX	*In vitro* and *in vivo*	MCF-7	Wang et al. (2020)
		Polymeric micelles	DOX and PD-L1 siRNA	*In vitro*	Hela, MDA-MB-231	Chen et al. (2019)
	Cancer cell membrane	Liposomes	BMS-202 and RA-V	*In vitro* and *in vivo*	CT26	Yao et al. (2022)

## 6 Summary and discussion

The application of the NCDS in combination therapy with different therapeutic agents represents enormous potential in cancer therapy. The performance of nanocarriers is primarily determined by the material properties of the carriers. Selecting an appropriate carrier can enhance the solubility of drugs, prolong the circulation time of drugs in the blood, and control their release. This can lead to a reduction in the distribution of drugs in healthy tissues and an increase in their accumulation in tumor tissues, thereby reducing adverse reactions and improving therapeutic efficacy. By facilitating the concurrent administration of therapeutic agents, the NCDS enables precision modulation of both the temporal and spatial distribution of these agents within tumor tissues, culminating in a therapeutic approach that is markedly more efficacious while concurrently mitigating collateral toxicities. Comparative analyses reveal that the NCDS offers superior pharmacokinetic attributes relative to conventional polypharmacy approaches. Nanocarriers, including lipid-based NPs, polymeric NPs, dendrimers, and inorganic systems, gain much attention for combination therapy. The principal obstacles impeding the NCDS are rooted in the complexities and technical hurdles manifested during clinical translational phases, which include maintaining a consistent ratio of the therapeutic agents, fine-tuning the pharmacodynamics of drug release, and achieving precise tumor targeting. Moreover, the engineering of co-delivery systems capable of stably incorporating and delivering multiple pharmacological agents to tumor tissues remains a formidable challenge, particularly when contemplating scale-up for mass production. The strategy of agent release—whether asynchronous or synchronous—must be tailored to the specificities of the treatment protocol. To this end, tumor microenvironment-responsive NCDSs are being meticulously engineered to ensure controlled release kinetics. Looking ahead, the vanguard of NCDS design is likely to be characterized by multifunctional carriers that are adept at navigating the labyrinthine tumor microenvironment, thereby enhancing the synergism of therapeutic modalities and ameliorating the intrinsic limitations associated with drug delivery. Nevertheless, the sophistication of such multi-responsive systems introduces commensurate complexities in quality assurance during the manufacturing process. In the context of targeted NCDS, exploratory efforts have been directed toward nanoparticles conjugated with tumor-homing entities such as specific ligands, antibodies, and saccharides, or cell membranes. It is imperative to consider the potential for oncogenic genetic material within the camouflage of cell membrane-mimetic nanoparticles. Although the co-delivery systems are generally non-toxic, flexible, biocompatible, biodegradable, and non-immunogenic for systemic administrations, as well as possessing high intracellular uptake, the clinically applied NCDS is still rarely available in the market. Only three distinct liposomal co-drug systems: CPX-1 (comprising fluorouracil and irinotecan), CPX-351 (comprising cytarabine and daunorubicin), and CPX-571 (comprising irinotecan and cisplatin) have been developed by Celator Pharmaceuticals Inc. (Dicko et al., 2010; Hourigan and Karp, 2010). Despite the formidable challenges and the nascent stage of NCDS application, these systems have demonstrated proficiency in overcoming MDR, inducing cell apoptosis in cancer therapy, limiting tumor metastasis in cancer, inhibiting angiogenesis, inducing cell ferroptosis, enhancing antitumor immunity, and so on. As advancements in material sciences and biomedical research continue to surge, it is anticipated that the impediments currently facing the NCDS will be surmounted.
